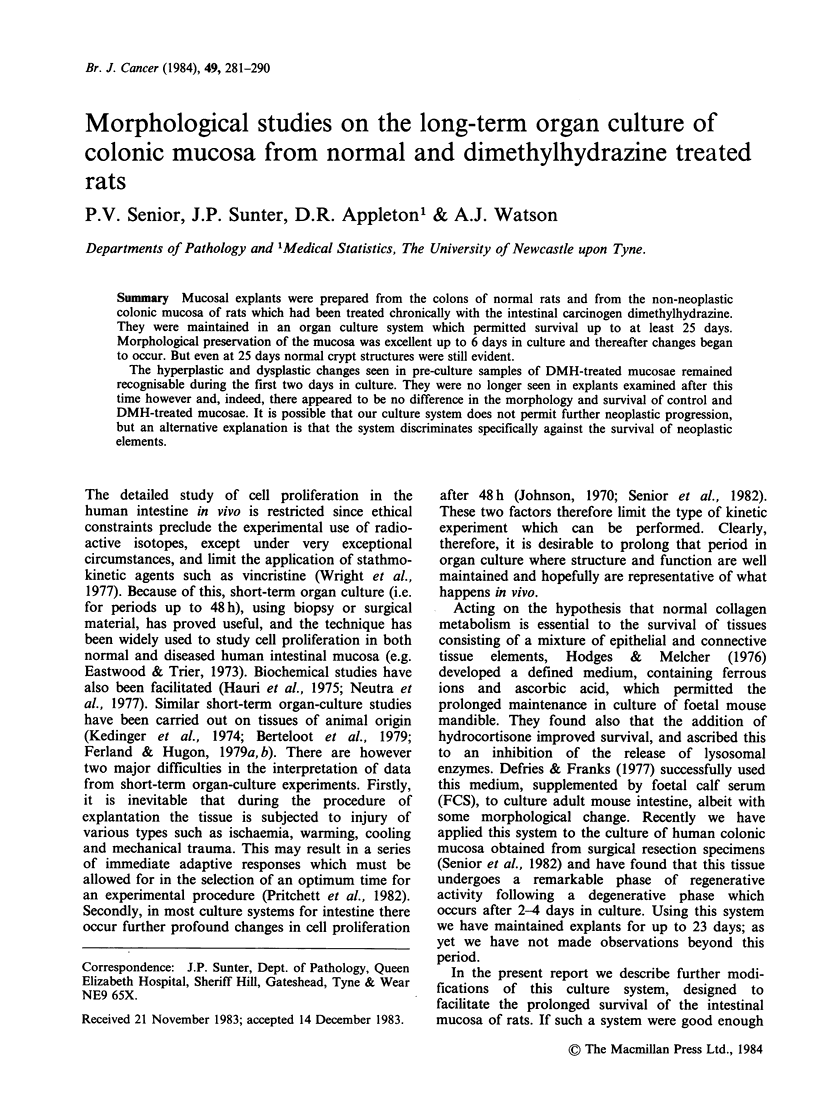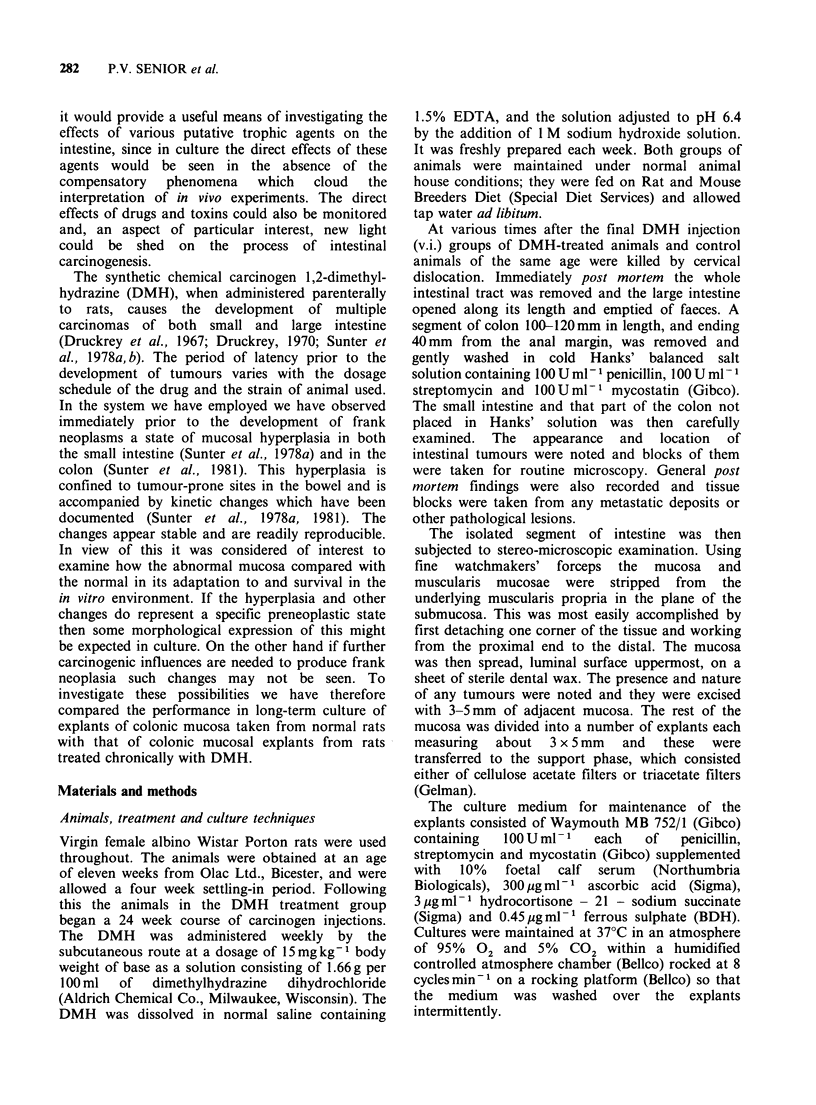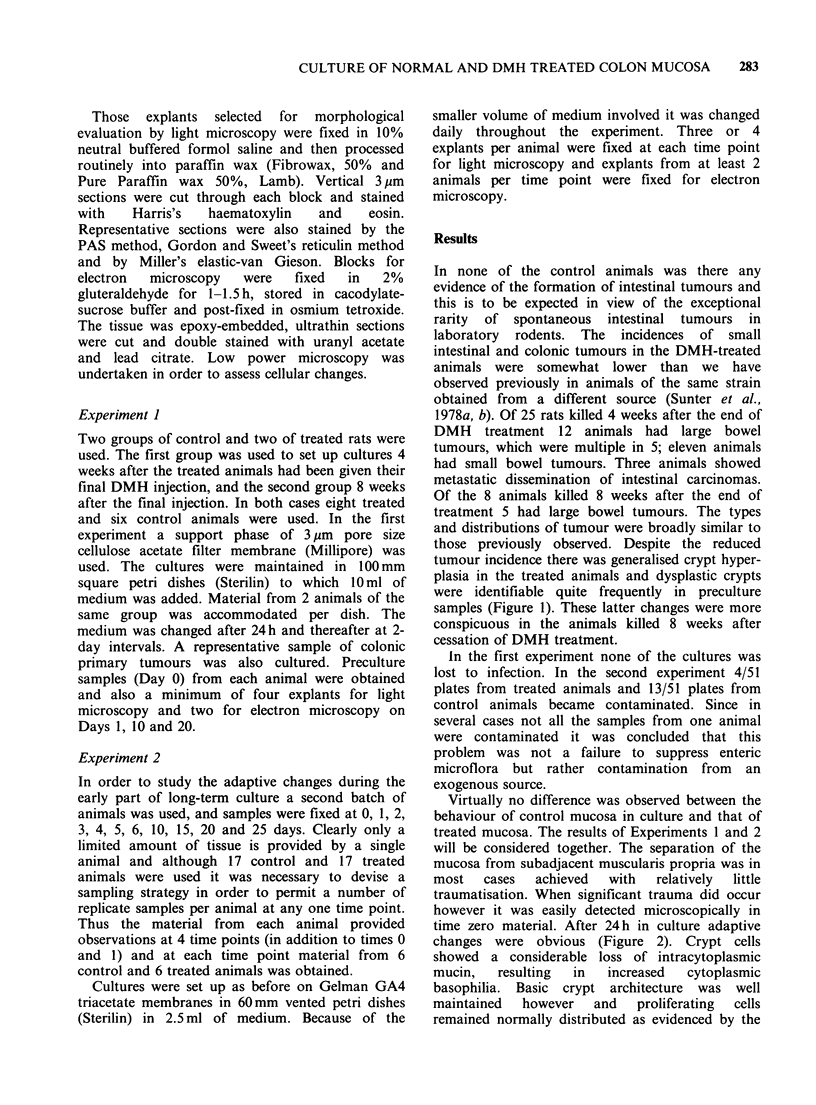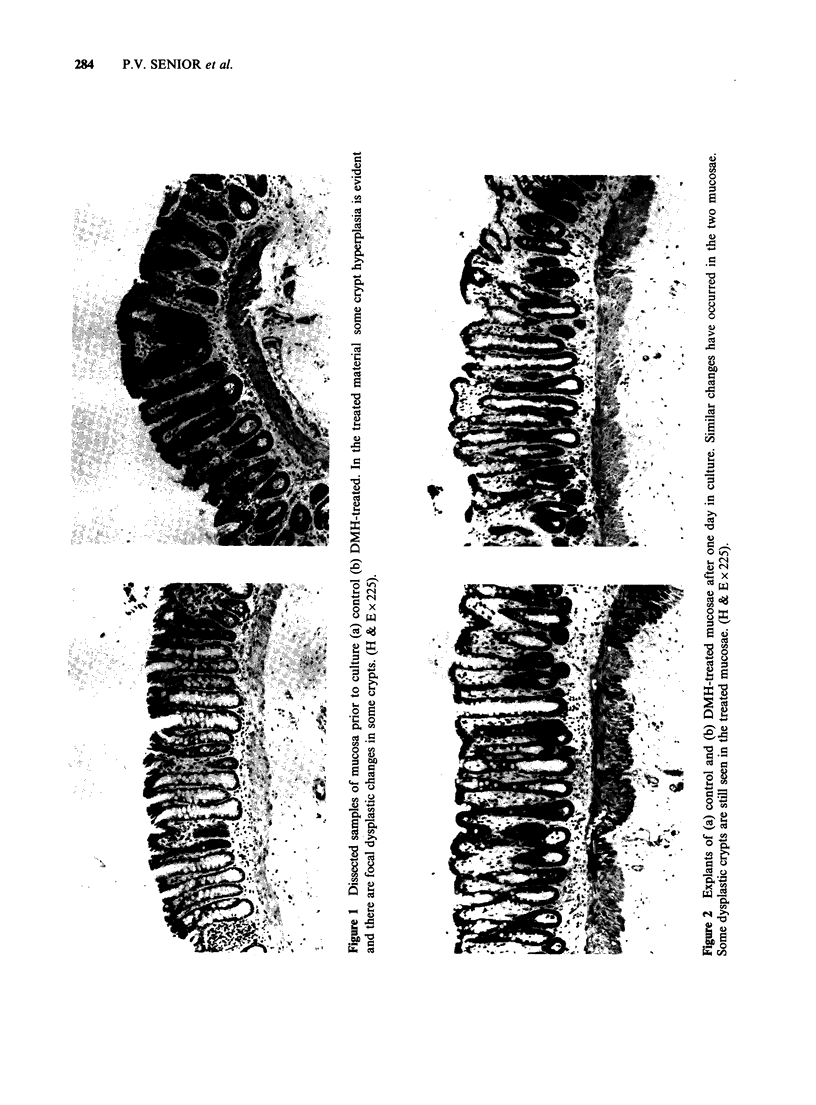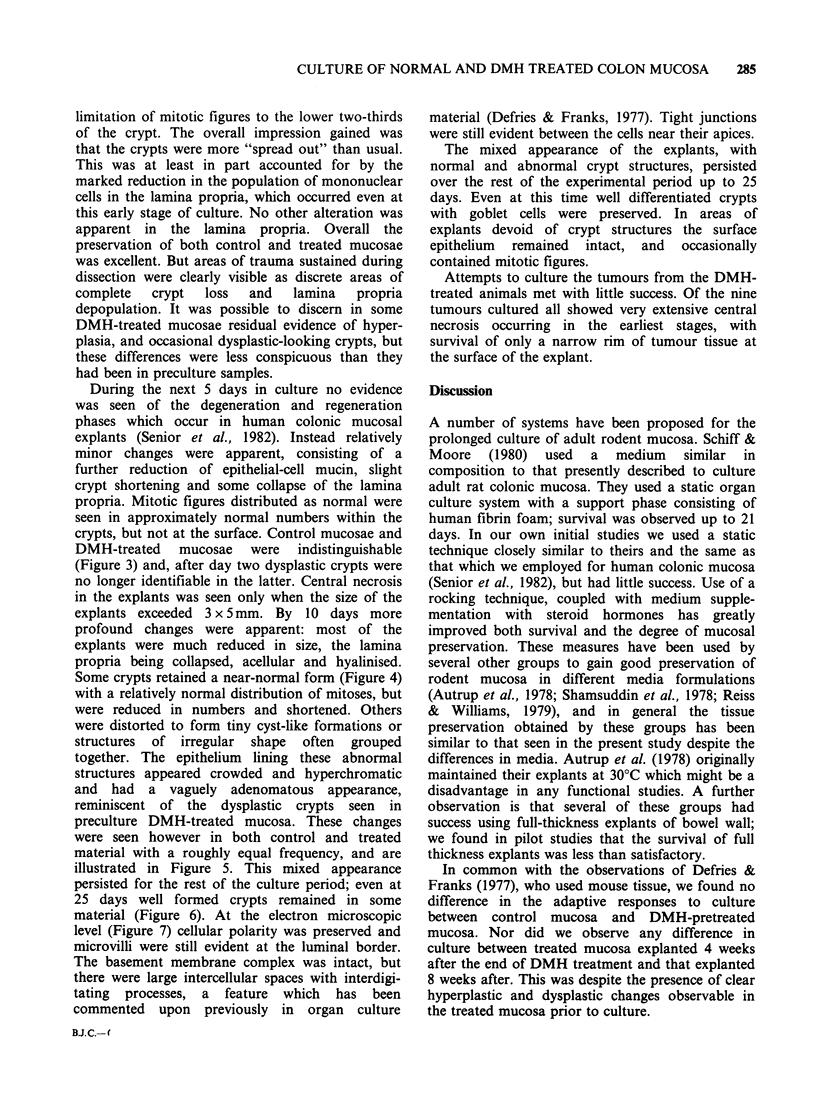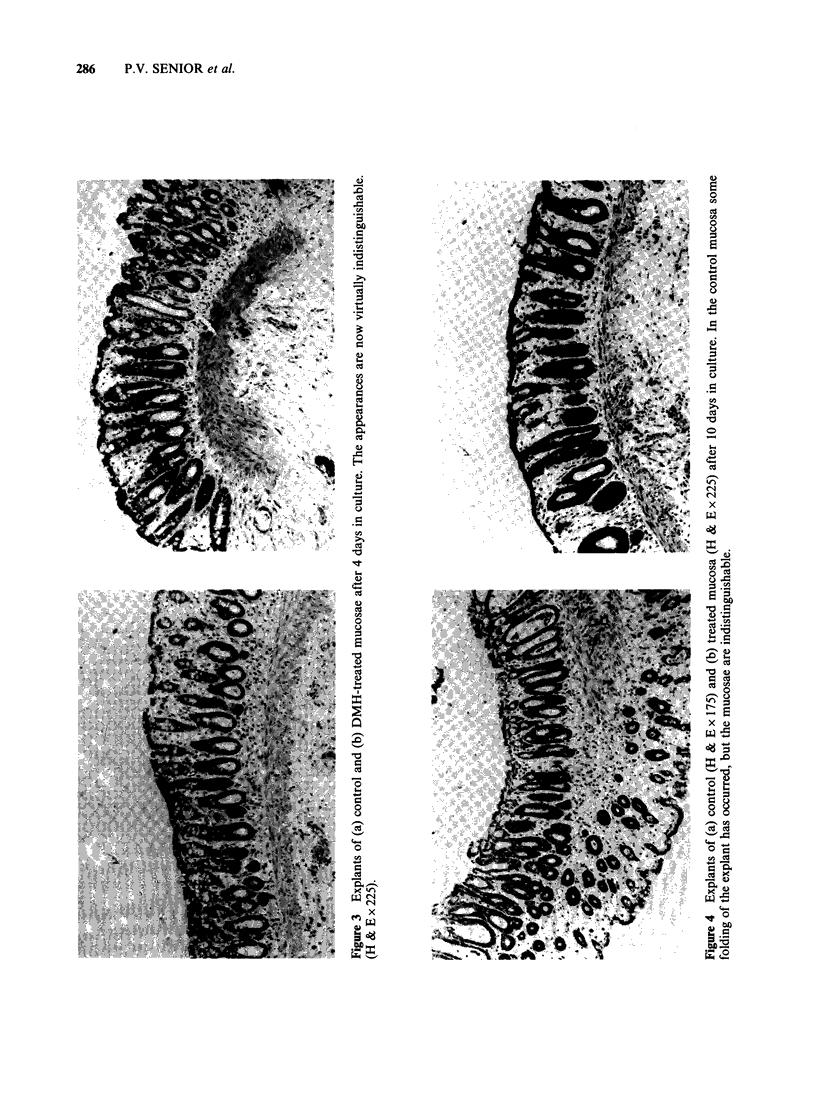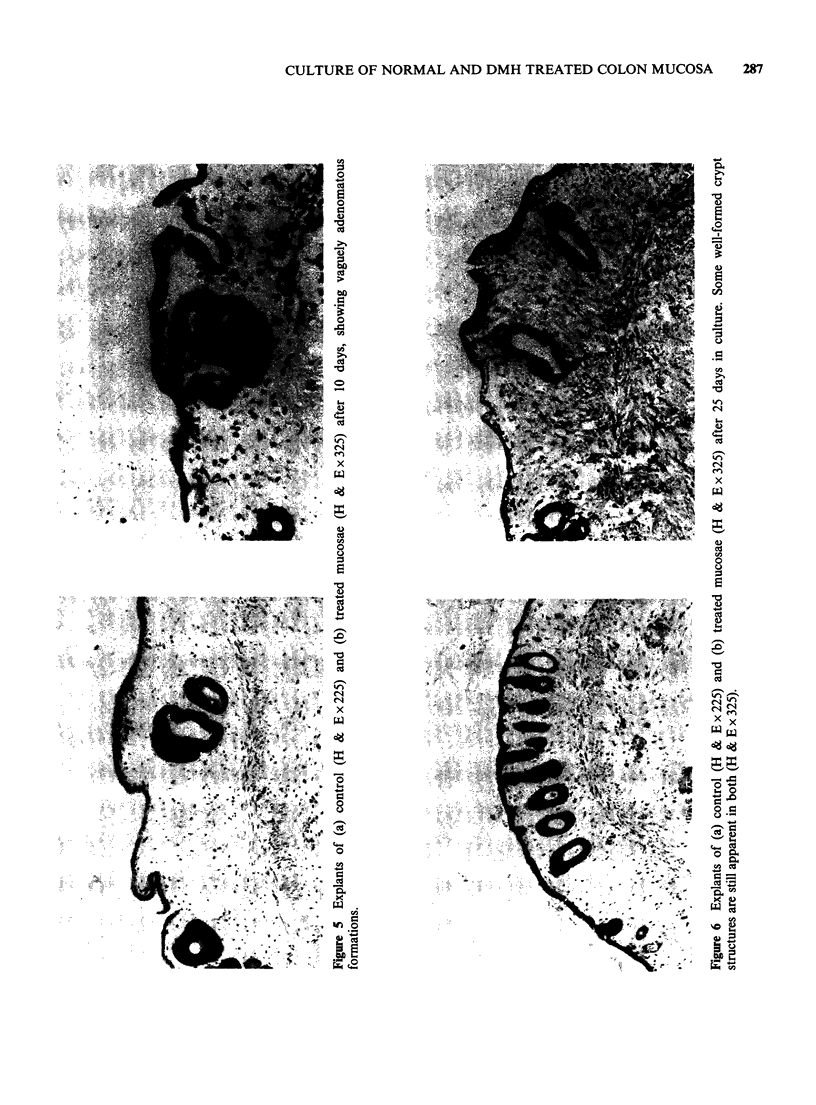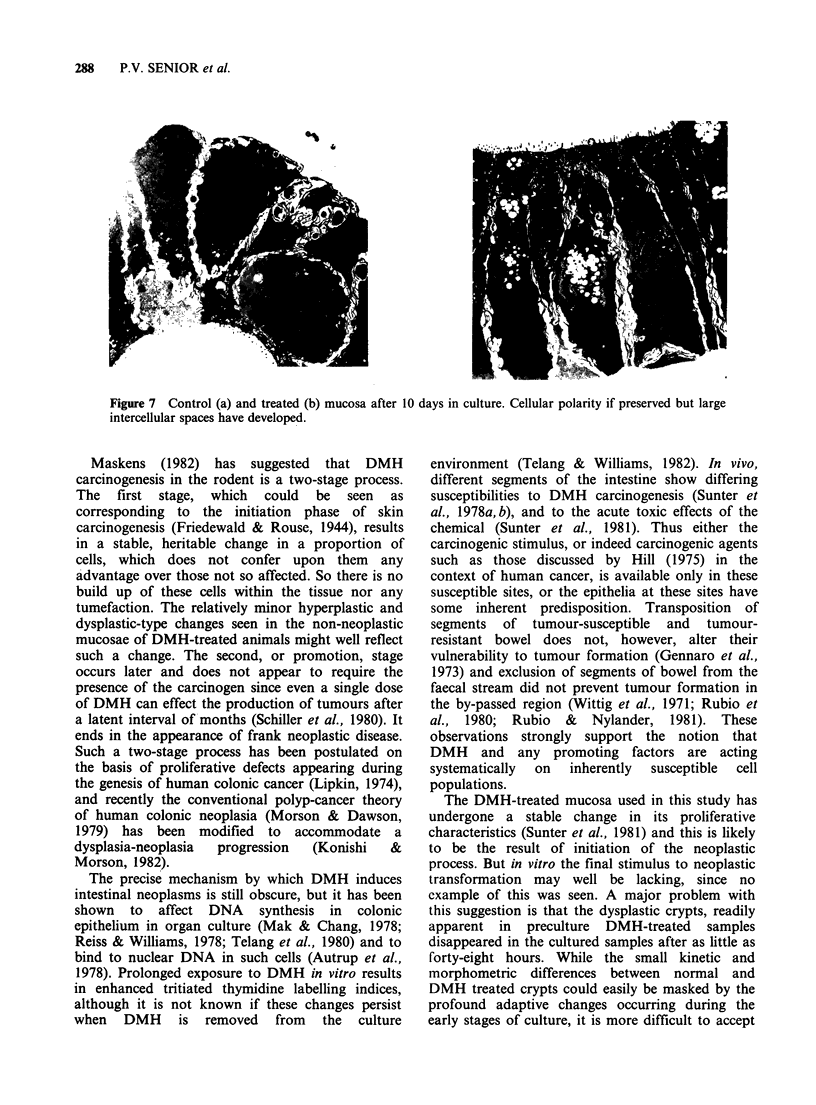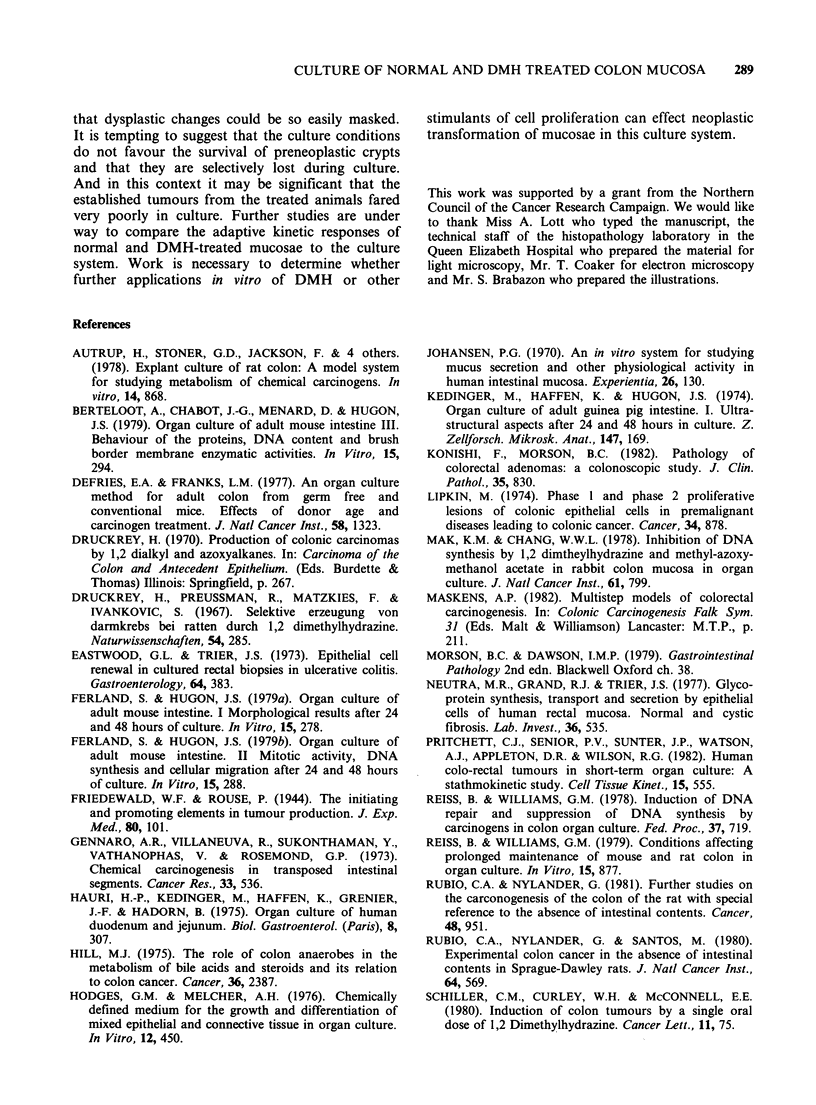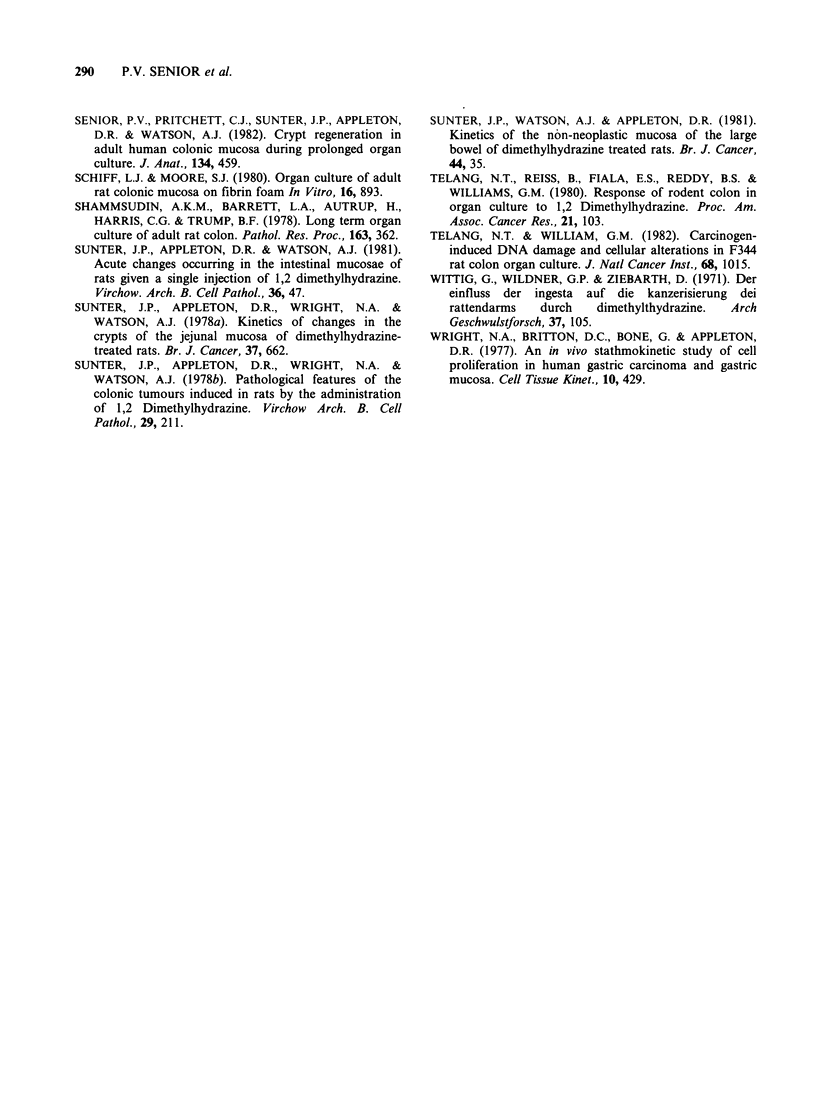# Morphological studies on the long-term organ culture of colonic mucosa from normal and dimethylhydrazine treated rats.

**DOI:** 10.1038/bjc.1984.45

**Published:** 1984-03

**Authors:** P. V. Senior, J. P. Sunter, D. R. Appleton, A. J. Watson

## Abstract

**Images:**


*Br. J. Cancer* (1984), **49**, 281–290

### Morphological studies on the long-term organ culture of colonic mucosa from normal and dimethylhydrazine treated rats

P.V. Senior, J.P. Sunter, D.R. Appleton[1] & A.J. Watson

*Departments of Pathology and [1]Medical Statistics, The University of Newcastle upon Tyne.*

**Summary** Mucosal explants were prepared from the colons of normal rats and from the non-neoplastic colonic mucosa of rats which had been treated chronically with the intestinal carcinogen dimethylhydrazine. They were maintained in an organ culture system which permitted survival up to at least 25 days. Morphological preservation of the mucosa was excellent up to 6 days in culture and thereafter changes began to occur. But even at 25 days normal crypt structures were still evident.

The hyperplastic and dysplastic changes seen in pre-culture samples of DMH-treated mucosae remained recognisable during the first two days in culture. They were no longer seen in explants examined after this time however and, indeed, there appeared to be no difference in the morphology and survival of control and DMH-treated mucosae. It is possible that our culture system does not permit further neoplastic progression, but an alternative explanation is that the system discriminates specifically against the survival of neoplastic elements.

The detailed study of cell proliferation in the human intestine *in vivo* is restricted since ethical constraints preclude the experimental use of radioactive isotopes, except under very exceptional circumstances, and limit the application of stathmokinetic agents such as vincristine (Wright *et al.*, 1977). Because of this, short-term organ culture (i.e. for periods up to 48 h), using biopsy or surgical material, has proved useful, and the technique has been widely used to study cell proliferation in both normal and diseased human intestinal mucosa (e.g. Eastwood & Trier, 1973). Biochemical studies have also been facilitated (Hauri *et al.*, 1975; Neutra *et al.*, 1977). Similar short-term organ-culture studies have been carried out on tissues of animal origin (Kedinger *et al.*, 1974; Berteloot *et al.*, 1979; Ferland & Hugon, 1979*a, b*). There are however two major difficulties in the interpretation of data from short-term organ-culture experiments. Firstly, it is inevitable that during the procedure of explantation the tissue is subjected to injury of various types such as ischaemia, warming, cooling and mechanical trauma. This may result in a series of immediate adaptive responses which must be allowed for in the selection of an optimum time for an experimental procedure (Pritchett *et al.*, 1982). Secondly, in most culture systems for intestine there occur further profound changes in cell proliferation after 48 h (Johnson, 1970; Senior *et al.*, 1982). These two factors therefore limit the type of kinetic experiment which can be performed. Clearly, therefore, it is desirable to prolong that period in organ culture where structure and function are well maintained and hopefully are representative of what happens *in vivo*.

Acting on the hypothesis that normal collagen metabolism is essential to the survival of tissues consisting of a mixture of epithelial and connective tissue elements, Hodges & Melcher (1976) developed a defined medium, containing ferrous ions and ascorbic acid, which permitted the prolonged maintenance in culture of foetal mouse mandible. They found also that the addition of hydrocortisone improved survival, and ascribed this to an inhibition of the release of lysosomal enzymes. Defries & Franks (1977) successfully used this medium, supplemented by foetal calf serum (FCS), to culture adult mouse intestine, albeit with some morphological change. Recently we have applied this system to the culture of human colonic mucosa obtained from surgical resection specimens (Senior *et al.*, 1982) and have found that this tissue undergoes a remarkable phase of regenerative activity following a degenerative phase which occurs after 2–4 days in culture. Using this system we have maintained explants for up to 23 days; as yet we have not made observations beyond this period.

In the present report we describe further modifications of this culture system, designed to facilitate the prolonged survival of the intestinal mucosa of rats. If such a system were good enough

Correspondence: J.P. Sunter, Dept. of Pathology, Queen Elizabeth Hospital, Sheriff Hill, Gateshead, Tyne & Wear NE9 6SX.

Received 21 November 1983; accepted 14 December 1983.

© The Macmillan Press Ltd., 1984

it would provide a useful means of investigating the effects of various putative trophic agents on the intestine, since in culture the direct effects of these agents would be seen in the absence of the compensatory phenomena which cloud the interpretation of *in vivo* experiments. The direct effects of drugs and toxins could also be monitored and, an aspect of particular interest, new light could be shed on the process of intestinal carcinogenesis.

The synthetic chemical carcinogen 1,2-dimethyl-hydrazine (DMH), when administered parenterally to rats, causes the development of multiple carcinomas of both small and large intestine (Druckrey *et al.*, 1967; Druckrey, 1970; Sunter *et al.*, 1978*a,b*). The period of latency prior to the development of tumours varies with the dosage schedule of the drug and the strain of animal used. In the system we have employed we have observed immediately prior to the development of frank neoplasms a state of mucosal hyperplasia in both the small intestine (Sunter *et al.*, 1978*a*) and in the colon (Sunter *et al.*, 1981). This hyperplasia is confined to tumour-prone sites in the bowel and is accompanied by kinetic changes which have been documented (Sunter *et al.*, 1978*a*, 1981). The changes appear stable and are readily reproducible. In view of this it was considered of interest to examine how the abnormal mucosa compared with the normal in its adaptation to and survival in the *in vitro* environment. If the hyperplasia and other changes do represent a specific preneoplastic state then some morphological expression of this might be expected in culture. On the other hand if further carcinogenic influences are needed to produce frank neoplasia such changes may not be seen. To investigate these possibilities we have therefore compared the performance in long-term culture of explants of colonic mucosa taken from normal rats with that of colonic mucosal explants from rats treated chronically with DMH.

### Materials and methods

### *Animals, treatment and culture techniques*

Virgin female albino Wistar Porton rats were used throughout. The animals were obtained at an age of eleven weeks from Olac Ltd., Bicester, and were allowed a four week settling-in period. Following this the animals in the DMH treatment group began a 24 week course of carcinogen injections. The DMH was administered weekly by the subcutaneous route at a dosage of $15\,mg\,kg^{-1}$ body weight of base as a solution consisting of 1.66 g per 100 ml of dimethylhydrazine dihydrochloride (Aldrich Chemical Co., Milwaukee, Wisconsin). The DMH was dissolved in normal saline containing 1.5% EDTA, and the solution adjusted to pH 6.4 by the addition of 1 M sodium hydroxide solution. It was freshly prepared each week. Both groups of animals were maintained under normal animal house conditions; they were fed on Rat and Mouse Breeders Diet (Special Diet Services) and allowed tap water *ad libitum*.

At various times after the final DMH injection (v.i.) groups of DMH-treated animals and control animals of the same age were killed by cervical dislocation. Immediately *post mortem* the whole intestinal tract was removed and the large intestine opened along its length and emptied of faeces. A segment of colon 100–120 mm in length, and ending 40 mm from the anal margin, was removed and gently washed in cold Hanks' balanced salt solution containing $100\,U\,ml^{-1}$ penicillin, $100\,U\,ml^{-1}$ streptomycin and $100\,U\,ml^{-1}$ mycostatin (Gibco). The small intestine and that part of the colon not placed in Hanks' solution was then carefully examined. The appearance and location of intestinal tumours were noted and blocks of them were taken for routine microscopy. General *post mortem* findings were also recorded and tissue blocks were taken from any metastatic deposits or other pathological lesions.

The isolated segment of intestine was then subjected to stereo-microscopic examination. Using fine watchmakers' forceps the mucosa and muscularis mucosae were stripped from the underlying muscularis propria in the plane of the submucosa. This was most easily accomplished by first detaching one corner of the tissue and working from the proximal end to the distal. The mucosa was then spread, luminal surface uppermost, on a sheet of sterile dental wax. The presence and nature of any tumours were noted and they were excised with 3–5 mm of adjacent mucosa. The rest of the mucosa was divided into a number of explants each measuring about $3\times5$ mm and these were transferred to the support phase, which consisted either of cellulose acetate filters or triacetate filters (Gelman).

The culture medium for maintenance of the explants consisted of Waymouth MB 752/1 (Gibco) containing $100\,U\,ml^{-1}$ each of penicillin, streptomycin and mycostatin (Gibco) supplemented with 10% foetal calf serum (Northumbria Biologicals), $300\,\mu g\,ml^{-1}$ ascorbic acid (Sigma), $3\,\mu g\,ml^{-1}$ hydrocortisone – 21 – sodium succinate (Sigma) and $0.45\,\mu g\,ml^{-1}$ ferrous sulphate (BDH). Cultures were maintained at 37°C in an atmosphere of 95% $O_2$ and 5% $CO_2$ within a humidified controlled atmosphere chamber (Bellco) rocked at 8 cycles $min^{-1}$ on a rocking platform (Bellco) so that the medium was washed over the explants intermittently.

Those explants selected for morphological evaluation by light microscopy were fixed in 10% neutral buffered formol saline and then processed routinely into paraffin wax (Fibrowax, 50% and Pure Paraffin wax 50%, Lamb). Vertical 3 $\mu$m sections were cut through each block and stained with Harris's haematoxylin and eosin. Representative sections were also stained by the PAS method, Gordon and Sweet's reticulin method and by Miller's elastic-van Gieson. Blocks for electron microscopy were fixed in 2% gluteraldehyde for 1–1.5 h, stored in cacodylate-sucrose buffer and post-fixed in osmium tetroxide. The tissue was epoxy-embedded, ultrathin sections were cut and double stained with uranyl acetate and lead citrate. Low power microscopy was undertaken in order to assess cellular changes.

*Experiment 1*

Two groups of control and two of treated rats were used. The first group was used to set up cultures 4 weeks after the treated animals had been given their final DMH injection, and the second group 8 weeks after the final injection. In both cases eight treated and six control animals were used. In the first experiment a support phase of 3 $\mu$m pore size cellulose acetate filter membrane (Millipore) was used. The cultures were maintained in 100 mm square petri dishes (Sterilin) to which 10 ml of medium was added. Material from 2 animals of the same group was accommodated per dish. The medium was changed after 24 h and thereafter at 2-day intervals. A representative sample of colonic primary tumours was also cultured. Preculture samples (Day 0) from each animal were obtained and also a minimum of four explants for light microscopy and two for electron microscopy on Days 1, 10 and 20.

*Experiment 2*

In order to study the adaptive changes during the early part of long-term culture a second batch of animals was used, and samples were fixed at 0, 1, 2, 3, 4, 5, 6, 10, 15, 20 and 25 days. Clearly only a limited amount of tissue is provided by a single animal and although 17 control and 17 treated animals were used it was necessary to devise a sampling strategy in order to permit a number of replicate samples per animal at any one time point. Thus the material from each animal provided observations at 4 time points (in addition to times 0 and 1) and at each time point material from 6 control and 6 treated animals was obtained.

Cultures were set up as before on Gelman GA4 triacetate membranes in 60 mm vented petri dishes (Sterilin) in 2.5 ml of medium. Because of the smaller volume of medium involved it was changed daily throughout the experiment. Three or 4 explants per animal were fixed at each time point for light microscopy and explants from at least 2 animals per time point were fixed for electron microscopy.

**Results**

In none of the control animals was there any evidence of the formation of intestinal tumours and this is to be expected in view of the exceptional rarity of spontaneous intestinal tumours in laboratory rodents. The incidences of small intestinal and colonic tumours in the DMH-treated animals were somewhat lower than we have observed previously in animals of the same strain obtained from a different source (Sunter *et al.*, 1978*a*, *b*). Of 25 rats killed 4 weeks after the end of DMH treatment 12 animals had large bowel tumours, which were multiple in 5; eleven animals had small bowel tumours. Three animals showed metastatic dissemination of intestinal carcinomas. Of the 8 animals killed 8 weeks after the end of treatment 5 had large bowel tumours. The types and distributions of tumour were broadly similar to those previously observed. Despite the reduced tumour incidence there was generalised crypt hyperplasia in the treated animals and dysplastic crypts were identifiable quite frequently in preculture samples (Figure 1). These latter changes were more conspicuous in the animals killed 8 weeks after cessation of DMH treatment.

In the first experiment none of the cultures was lost to infection. In the second experiment 4/51 plates from treated animals and 13/51 plates from control animals became contaminated. Since in several cases not all the samples from one animal were contaminated it was concluded that this problem was not a failure to suppress enteric microflora but rather contamination from an exogenous source.

Virtually no difference was observed between the behaviour of control mucosa in culture and that of treated mucosa. The results of Experiments 1 and 2 will be considered together. The separation of the mucosa from subadjacent muscularis propria was in most cases achieved with relatively little traumatisation. When significant trauma did occur however it was easily detected microscopically in time zero material. After 24 h in culture adaptive changes were obvious (Figure 2). Crypt cells showed a considerable loss of intracytoplasmic mucin, resulting in increased cytoplasmic basophilia. Basic crypt architecture was well maintained however and proliferating cells remained normally distributed as evidenced by the

**Figure 1** Dissected samples of mucosa prior to culture (a) control (b) DMH-treated. In the treated material some crypt hyperplasia is evident and there are focal dysplastic changes in some crypts. (H & E × 225).

**Figure 2** Explants of (a) control and (b) DMH-treated mucosae after one day in culture. Similar changes have occurred in the two mucosae. Some dysplastic crypts are still seen in the treated mucosae. (H & E × 225).

limitation of mitotic figures to the lower two-thirds of the crypt. The overall impression gained was that the crypts were more "spread out" than usual. This was at least in part accounted for by the marked reduction in the population of mononuclear cells in the lamina propria, which occurred even at this early stage of culture. No other alteration was apparent in the lamina propria. Overall the preservation of both control and treated mucosae was excellent. But areas of trauma sustained during dissection were clearly visible as discrete areas of complete crypt loss and lamina propria depopulation. It was possible to discern in some DMH-treated mucosae residual evidence of hyperplasia, and occasional dysplastic-looking crypts, but these differences were less conspicuous than they had been in preculture samples.

During the next 5 days in culture no evidence was seen of the degeneration and regeneration phases which occur in human colonic mucosal explants (Senior *et al.*, 1982). Instead relatively minor changes were apparent, consisting of a further reduction of epithelial-cell mucin, slight crypt shortening and some collapse of the lamina propria. Mitotic figures distributed as normal were seen in approximately normal numbers within the crypts, but not at the surface. Control mucosae and DMH-treated mucosae were indistinguishable (Figure 3) and, after day two dysplastic crypts were no longer identifiable in the latter. Central necrosis in the explants was seen only when the size of the explants exceeded 3 x 5 mm. By 10 days more profound changes were apparent: most of the explants were much reduced in size, the lamina propria being collapsed, acellular and hyalinised. Some crypts retained a near-normal form (Figure 4) with a relatively normal distribution of mitoses, but were reduced in numbers and shortened. Others were distorted to form tiny cyst-like formations or structures of irregular shape often grouped together. The epithelium lining these abnormal structures appeared crowded and hyperchromatic and had a vaguely adenomatous appearance, reminiscent of the dysplastic crypts seen in preculture DMH-treated mucosa. These changes were seen however in both control and treated material with a roughly equal frequency, and are illustrated in Figure 5. This mixed appearance persisted for the rest of the culture period; even at 25 days well formed crypts remained in some material (Figure 6). At the electron microscopic level (Figure 7) cellular polarity was preserved and microvilli were still evident at the luminal border. The basement membrane complex was intact, but there were large intercellular spaces with interdigitating processes, a feature which has been commented upon previously in organ culture material (Defries & Franks, 1977). Tight junctions were still evident between the cells near their apices.

The mixed appearance of the explants, with normal and abnormal crypt structures, persisted over the rest of the experimental period up to 25 days. Even at this time well differentiated crypts with goblet cells were preserved. In areas of explants devoid of crypt structures the surface epithelium remained intact, and occasionally contained mitotic figures.

Attempts to culture the tumours from the DMH-treated animals met with little success. Of the nine tumours cultured all showed very extensive central necrosis occurring in the earliest stages, with survival of only a narrow rim of tumour tissue at the surface of the explant.

### Discussion

A number of systems have been proposed for the prolonged culture of adult rodent mucosa. Schiff & Moore (1980) used a medium similar in composition to that presently described to culture adult rat colonic mucosa. They used a static organ culture system with a support phase consisting of human fibrin foam; survival was observed up to 21 days. In our own initial studies we used a static technique closely similar to theirs and the same as that which we employed for human colonic mucosa (Senior *et al.*, 1982), but had little success. Use of a rocking technique, coupled with medium supplementation with steroid hormones has greatly improved both survival and the degree of mucosal preservation. These measures have been used by several other groups to gain good preservation of rodent mucosa in different media formulations (Autrup *et al.*, 1978; Shamsuddin *et al.*, 1978; Reiss & Williams, 1979), and in general the tissue preservation obtained by these groups has been similar to that seen in the present study despite the differences in media. Autrup *et al.* (1978) originally maintained their explants at 30°C which might be a disadvantage in any functional studies. A further observation is that several of these groups had success using full-thickness explants of bowel wall; we found in pilot studies that the survival of full thickness explants was less than satisfactory.

In common with the observations of Defries & Franks (1977), who used mouse tissue, we found no difference in the adaptive responses to culture between control mucosa and DMH-pretreated mucosa. Nor did we observe any difference in culture between treated mucosa explanted 4 weeks after the end of DMH treatment and that explanted 8 weeks after. This was despite the presence of clear hyperplastic and dysplastic changes observable in the treated mucosa prior to culture.

**Figure 7** Control (a) and treated (b) mucosa after 10 days in culture. Cellular polarity if preserved but large intercellular spaces have developed.

Maskens (1982) has suggested that DMH carcinogenesis in the rodent is a two-stage process. The first stage, which could be seen as corresponding to the initiation phase of skin carcinogenesis (Friedewald & Rouse, 1944), results in a stable, heritable change in a proportion of cells, which does not confer upon them any advantage over those not so affected. So there is no build up of these cells within the tissue nor any tumefaction. The relatively minor hyperplastic and dysplastic-type changes seen in the non-neoplastic mucosae of DMH-treated animals might well reflect such a change. The second, or promotion, stage occurs later and does not appear to require the presence of the carcinogen since even a single dose of DMH can effect the production of tumours after a latent interval of months (Schiller *et al.*, 1980). It ends in the appearance of frank neoplastic disease. Such a two-stage process has been postulated on the basis of proliferative defects appearing during the genesis of human colonic cancer (Lipkin, 1974), and recently the conventional polyp-cancer theory of human colonic neoplasia (Morson & Dawson, 1979) has been modified to accommodate a dysplasia-neoplasia progression (Konishi & Morson, 1982).

The precise mechanism by which DMH induces intestinal neoplasms is still obscure, but it has been shown to affect DNA synthesis in colonic epithelium in organ culture (Mak & Chang, 1978; Reiss & Williams, 1978; Telang *et al.*, 1980) and to bind to nuclear DNA in such cells (Autrup *et al.*, 1978). Prolonged exposure to DMH *in vitro* results in enhanced tritiated thymidine labelling indices, although it is not known if these changes persist when DMH is removed from the culture environment (Telang & Williams, 1982). *In vivo,* different segments of the intestine show differing susceptibilities to DMH carcinogenesis (Sunter *et al.*, 1978*a, b*), and to the acute toxic effects of the chemical (Sunter *et al.*, 1981). Thus either the carcinogenic stimulus, or indeed carcinogenic agents such as those discussed by Hill (1975) in the context of human cancer, is available only in these susceptible sites, or the epithelia at these sites have some inherent predisposition. Transposition of segments of tumour-susceptible and tumour-resistant bowel does not, however, alter their vulnerability to tumour formation (Gennaro *et al.*, 1973) and exclusion of segments of bowel from the faecal stream did not prevent tumour formation in the by-passed region (Wittig *et al.*, 1971; Rubio *et al.*, 1980; Rubio & Nylander, 1981). These observations strongly support the notion that DMH and any promoting factors are acting systematically on inherently susceptible cell populations.

The DMH-treated mucosa used in this study has undergone a stable change in its proliferative characteristics (Sunter *et al.*, 1981) and this is likely to be the result of initiation of the neoplastic process. But *in vitro* the final stimulus to neoplastic transformation may well be lacking, since no example of this was seen. A major problem with this suggestion is that the dysplastic crypts, readily apparent in preculture DMH-treated samples disappeared in the cultured samples after as little as forty-eight hours. While the small kinetic and morphometric differences between normal and DMH treated crypts could easily be masked by the profound adaptive changes occurring during the early stages of culture, it is more difficult to accept that dysplastic changes could be so easily masked. It is tempting to suggest that the culture conditions do not favour the survival of preneoplastic crypts and that they are selectively lost during culture. And in this context it may be significant that the established tumours from the treated animals fared very poorly in culture. Further studies are under way to compare the adaptive kinetic responses of normal and DMH-treated mucosae to the culture system. Work is necessary to determine whether further applications *in vitro* of DMH or other stimulants of cell proliferation can effect neoplastic transformation of mucosae in this culture system.

This work was supported by a grant from the Northern Council of the Cancer Research Campaign. We would like to thank Miss A. Lott who typed the manuscript, the technical staff of the histopathology laboratory in the Queen Elizabeth Hospital who prepared the material for light microscopy, Mr. T. Coaker for electron microscopy and Mr. S. Brabazon who prepared the illustrations.

**References**

AUTRUP, H., STONER, G.D., JACKSON, F. & 4 others. (1978). Explant culture of rat colon: A model system for studying metabolism of chemical carcinogens. *In vitro*, **14**, 868.

BERTELOOT, A., CHABOT, J.-G., MENARD, D. & HUGON, J.S. (1979). Organ culture of adult mouse intestine III. Behaviour of the proteins, DNA content and brush border membrane enzymatic activities. *In Vitro*, **15**, 294.

DEFRIES, E.A. & FRANKS, L.M. (1977). An organ culture method for adult colon from germ free and conventional mice. Effects of donor age and carcinogen treatment. *J. Natl Cancer Inst.*, **58**, 1323.

DRUCKREY, H. (1970). Production of colonic carcinomas by 1,2 dialkyl and azoxyalkanes. In: *Carcinoma of the Colon and Antecedent Epithelium*. (Eds. Burdette & Thomas) Illinois: Springfield, p. 267.

DRUCKREY, H., PREUSSMAN, R., MATZKIES, F. & IVANKOVIC, S. (1967). Selektive erzeugung von darmkrebs bei ratten durch 1,2 dimethylhydrazine. *Naturwissenschaften*, **54**, 285.

EASTWOOD, G.L. & TRIER, J.S. (1973). Epithelial cell renewal in cultured rectal biopsies in ulcerative colitis. *Gastroenterology*, **64**, 383.

FERLAND, S. & HUGON, J.S. (1979*a*). Organ culture of adult mouse intestine. I Morphological results after 24 and 48 hours of culture. *In Vitro*, **15**, 278.

FERLAND, S. & HUGON, J.S. (1979*b*). Organ culture of adult mouse intestine. II Mitotic activity, DNA synthesis and cellular migration after 24 and 48 hours of culture. *In Vitro*, **15**, 288.

FRIEDEWALD, W.F. & ROUSE, P. (1944). The initiating and promoting elements in tumour production. *J. Exp. Med.*, **80**, 101.

GENNARO, A.R., VILLANEUVA, R., SUKONTHAMAN, Y., VATHANOPHAS, V. & ROSEMOND, G.P. (1973). Chemical carcinogenesis in transposed intestinal segments. *Cancer Res.*, **33**, 536.

HAURI, H.-P., KEDINGER, M., HAFFEN, K., GRENIER, J.-F. & HADORN, B. (1975). Organ culture of human duodenum and jejunum. *Biol. Gastroenterol. (Paris)*, **8**, 307.

HILL, M.J. (1975). The role of colon anaerobes in the metabolism of bile acids and steroids and its relation to colon cancer. *Cancer*, **36**, 2387.

HODGES, G.M. & MELCHER, A.H. (1976). Chemically defined medium for the growth and differentiation of mixed epithelial and connective tissue in organ culture. *In Vitro*, **12**, 450.

JOHANSEN, P.G. (1970). An *in vitro* system for studying mucus secretion and other physiological activity in human intestinal mucosa. *Experientia*, **26**, 130.

KEDINGER, M., HAFFEN, K. & HUGON, J.S. (1974). Organ culture of adult guinea pig intestine. I. Ultrastructural aspects after 24 and 48 hours in culture. *Z. Zellforsch. Mikrosk. Anat.*, **147**, 169.

KONISHI, F., MORSON, B.C. (1982). Pathology of colorectal adenomas: a colonoscopic study. *J. Clin. Pathol.*, **35**, 830.

LIPKIN, M. (1974). Phase 1 and phase 2 proliferative lesions of colonic epithelial cells in premalignant diseases leading to colonic cancer. *Cancer*, **34**, 878.

MAK, K.M. & CHANG, W.W.L. (1978). Inhibition of DNA synthesis by 1,2 dimtheylhydrazine and methyl-azoxymethanol acetate in rabbit colon mucosa in organ culture. *J. Natl Cancer Inst.*, **61**, 799.

MASKENS, A.P. (1982). Multistep models of colorectal carcinogenesis. In: *Colonic Carcinogenesis Falk Sym. 31* (Eds. Malt & Williamson) Lancaster: M.T.P., p. 211.

MORSON, B.C. & DAWSON, I.M.P. (1979). *Gastrointestinal Pathology* 2nd edn. Blackwell Oxford ch. 38.

NEUTRA, M.R., GRAND, R.J. & TRIER, J.S. (1977). Glycoprotein synthesis, transport and secretion by epithelial cells of human rectal mucosa. Normal and cystic fibrosis. *Lab. Invest.*, **36**, 535.

PRITCHETT, C.J., SENIOR, P.V., SUNTER, J.P., WATSON, A.J., APPLETON, D.R. & WILSON, R.G. (1982). Human colo-rectal tumours in short-term organ culture: A stathmokinetic study. *Cell Tissue Kinet.*, **15**, 555.

REISS, B. & WILLIAMS, G.M. (1978). Induction of DNA repair and suppression of DNA synthesis by carcinogens in colon organ culture. *Fed. Proc.*, **37**, 719.

REISS, B. & WILLIAMS, G.M. (1979). Conditions affecting prolonged maintenance of mouse and rat colon in organ culture. *In Vitro*, **15**, 877.

RUBIO, C.A. & NYLANDER, G. (1981). Further studies on the carconogenesis of the colon of the rat with special reference to the absence of intestinal contents. *Cancer*, **48**, 951.

RUBIO, C.A., NYLANDER, G. & SANTOS, M. (1980). Experimental colon cancer in the absence of intestinal contents in Sprague-Dawley rats. *J. Natl Cancer Inst.*, **64**, 569.

SCHILLER, C.M., CURLEY, W.H. & McCONNELL, E.E. (1980). Induction of colon tumours by a single oral dose of 1,2 Dimethylhydrazine. *Cancer Lett.*, **11**, 75.

SENIOR, P.V., PRITCHETT, C.J., SUNTER, J.P., APPLETON, D.R. & WATSON, A.J. (1982). Crypt regeneration in adult human colonic mucosa during prolonged organ culture. *J. Anat.*, **134,** 459.

SCHIFF, L.J. & MOORE, S.J. (1980). Organ culture of adult rat colonic mucosa on fibrin foam *In Vitro,* **16,** 893.

SHAMMSUDIN, A.K.M., BARRETT, L.A., AUTRUP, H., HARRIS, C.G. & TRUMP, B.F. (1978). Long term organ culture of adult rat colon. *Pathol. Res. Proc.*, **163,** 362.

SUNTER, J.P., APPLETON, D.R. & WATSON, A.J. (1981). Acute changes occurring in the intestinal mucosae of rats given a single injection of 1,2 dimethylhydrazine. *Virchow. Arch. B. Cell Pathol.*, **36,** 47.

SUNTER, J.P., APPLETON, D.R., WRIGHT, N.A. & WATSON, A.J. (1978*a*). Kinetics of changes in the crypts of the jejunal mucosa of dimethylhydrazine-treated rats. *Br. J. Cancer,* **37,** 662.

SUNTER, J.P., APPLETON, D.R., WRIGHT, N.A. & WATSON, A.J. (1978*b*). Pathological features of the colonic tumours induced in rats by the administration of 1,2 Dimethylhydrazine. *Virchow Arch. B. Cell Pathol.*, **29,** 211.

SUNTER, J.P., WATSON, A.J. & APPLETON, D.R. (1981). Kinetics of the nòn-neoplastic mucosa of the large bowel of dimethylhydrazine treated rats. *Br. J. Cancer,* **44,** 35.

TELANG, N.T., REISS, B., FIALA, E.S., REDDY, B.S. & WILLIAMS, G.M. (1980). Response of rodent colon in organ culture to 1,2 Dimethylhydrazine. *Proc. Am. Assoc. Cancer Res.*, **21,** 103.

TELANG, N.T. & WILLIAM, G.M. (1982). Carcinogen-induced DNA damage and cellular alterations in F344 rat colon organ culture. *J. Natl Cancer Inst.*, **68,** 1015.

WITTIG, G., WILDNER, G.P. & ZIEBARTH, D. (1971). Der einfluss der ingesta auf die kanzerisierung dei rattendarms durch dimethylthydrazine. *Arch Geschwulstforsch,* **37,** 105.

WRIGHT, N.A., BRITTON, D.C., BONE, G. & APPLETON, D.R. (1977). An *in vivo* stathmokinetic study of cell proliferation in human gastric carcinoma and gastric mucosa. *Cell Tissue Kinet.*, **10,** 429.